# Case Report: A neonatal case of severe congenital *Mycoplasma pneumoniae* pneumonia with atelectasis and macrolide resistance

**DOI:** 10.3389/fped.2025.1561097

**Published:** 2025-06-11

**Authors:** Dan Wang, Chunyan Yang, Daogang Qin, Jingcai Wang, Jing Zhao

**Affiliations:** Department of Pediatric, Liaocheng People’s Hospital, Liaocheng, Shandong, China

**Keywords:** *Mycoplasma pneumoniae* pneumonia, atelectasis, macrolide resistance, fluoroquinolone antibiotic, bronchoscopic lavage

## Abstract

Reports on congenital *Mycoplasma pneumoniae* (MP) pneumonia are rare in the medical literature. In this study, we presented a male neonatal case of severe congenital MP pneumonia complicated with atelectasis and macrolide resistance. Empirical treatment with conventional antibiotics proved ineffective and the ventilator could not be weaned. After undergoing five rounds of tracheal intubation, the neonate was successfully weaned off ventilator support after treatment with fluoroquinolone antibiotics combined with bronchoscopic lavage. The diagnoses were based on the early onset of respiratory distress, atypical pneumonia findings on pulmonary imaging, characteristic mucous plugs associated with MP observed during bronchoscopy, and positive MP nucleic acid and IgG antibodies in both the neonate and the mother. Importantly, this case report provides clinical evidence for the treatment of congenital MP infection with atelectasis and macrolide resistance by administering fluoroquinolones at appropriate dosages and durations, along with bronchoscopic lavage as an effective option to clear airway obstruction and improve pulmonary ventilation.

## Introduction

*Mycoplasma pneumoniae* (MP) is a common pathogen responsible for respiratory tract infections worldwide. Since 2023, MP has escalated to epidemic proportions in China, particularly affecting younger children, with more severe clinical manifestations and high resistance to macrolide antibiotics (1). To date, MP-associated neonatal pneumonia has been rarely reported, with congenital cases even less so. A review of English-language, peer-reviewed literature revealed only four reported cases of congenital MP pneumonia in neonates (2–5). To enrich our knowledge of this condition, we report a severe case of congenital MP infection in a neonate, complicated by atelectasis and macrolide resistance.

## Case report

**Figure 1:**
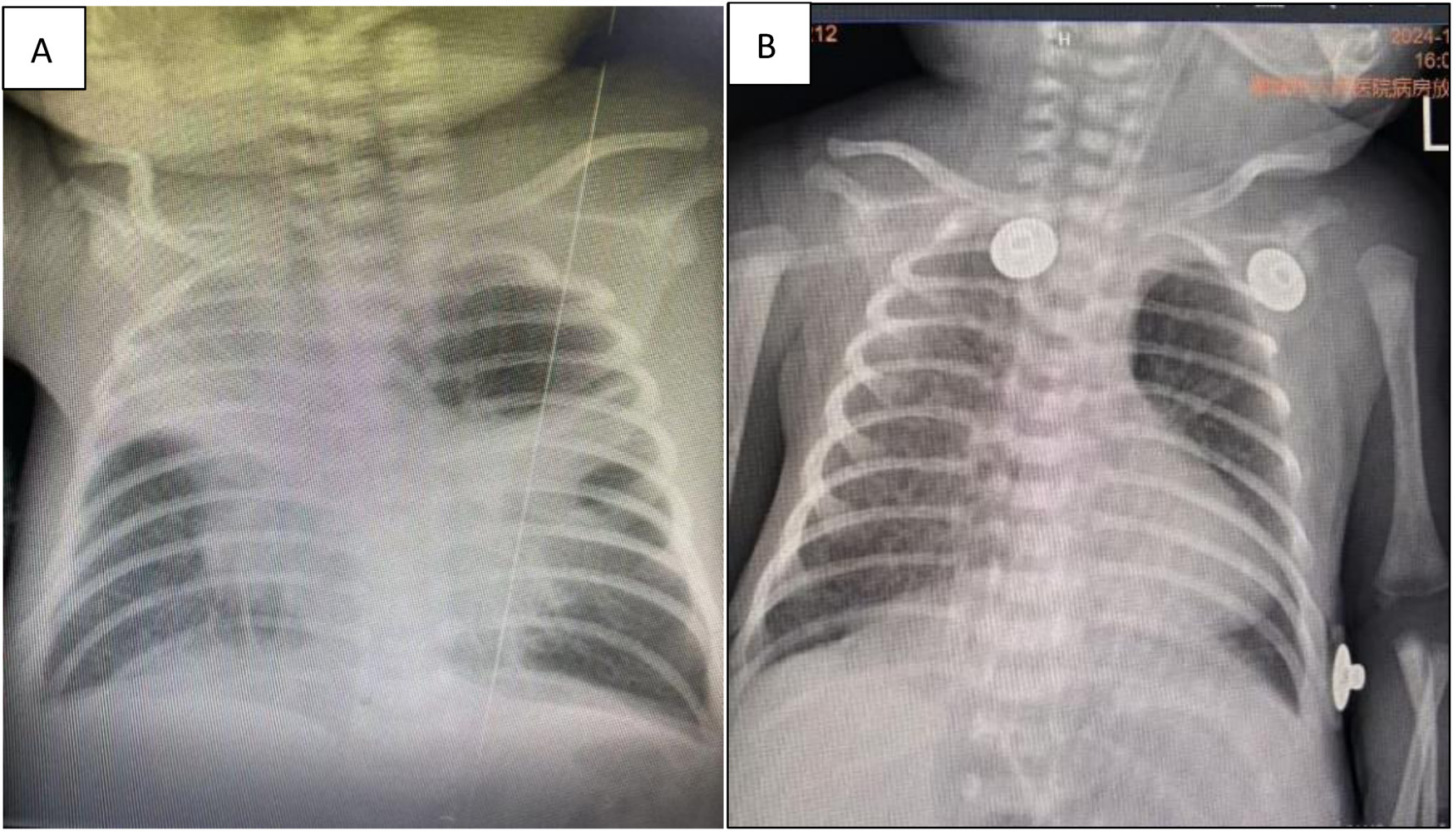
**(A)** Atelectasis of the left lung in chest radiographs. **(B)** Atelectasis after bronchoscopic lavage treatment during follow-up in chest radiographs.

A male neonate weighing 1,970 g at birth was delivered at 31 weeks of gestation by natural delivery after a 9-h rupture of membranes, without administration of lung maturation treatment. At birth, the infant exhibited cyanosis, weak crying, and signs of respiratory distress. His Apgar scores were 5 and 8 at 1 and 5 min, respectively. He was promptly transferred to the neonatal intensive care unit, where he required tracheal intubation and mechanical ventilation. Bovine surfactant (210 mg) was administered into the trachea according to standard protocol. Empirical antibiotic therapy with meropenem and penicillin was initiated for suspected infection. The neonate was successfully extubated on day 6 of life; however, he soon developed a respiratory distress syndrome. Nasal continuous positive airway pressure (CPAP) was commenced and used during the second round of tracheal intubation the next day. Given that the neonate’s serum mycoplasma IgG titer exceeded 300 U/ml, oral azithromycin was administered at a dose of 10 mg/kg and continued for 6 days. Despite treatment, extubation failed at the end of the azithromycin course due to left lung atelectasis, as confirmed by chest radiographs (Figure 1A). After a third tracheal intubation, the neonate was transferred to our department on day 13 of life with ventilatory support via a transport ventilator.

**Figure 2:**
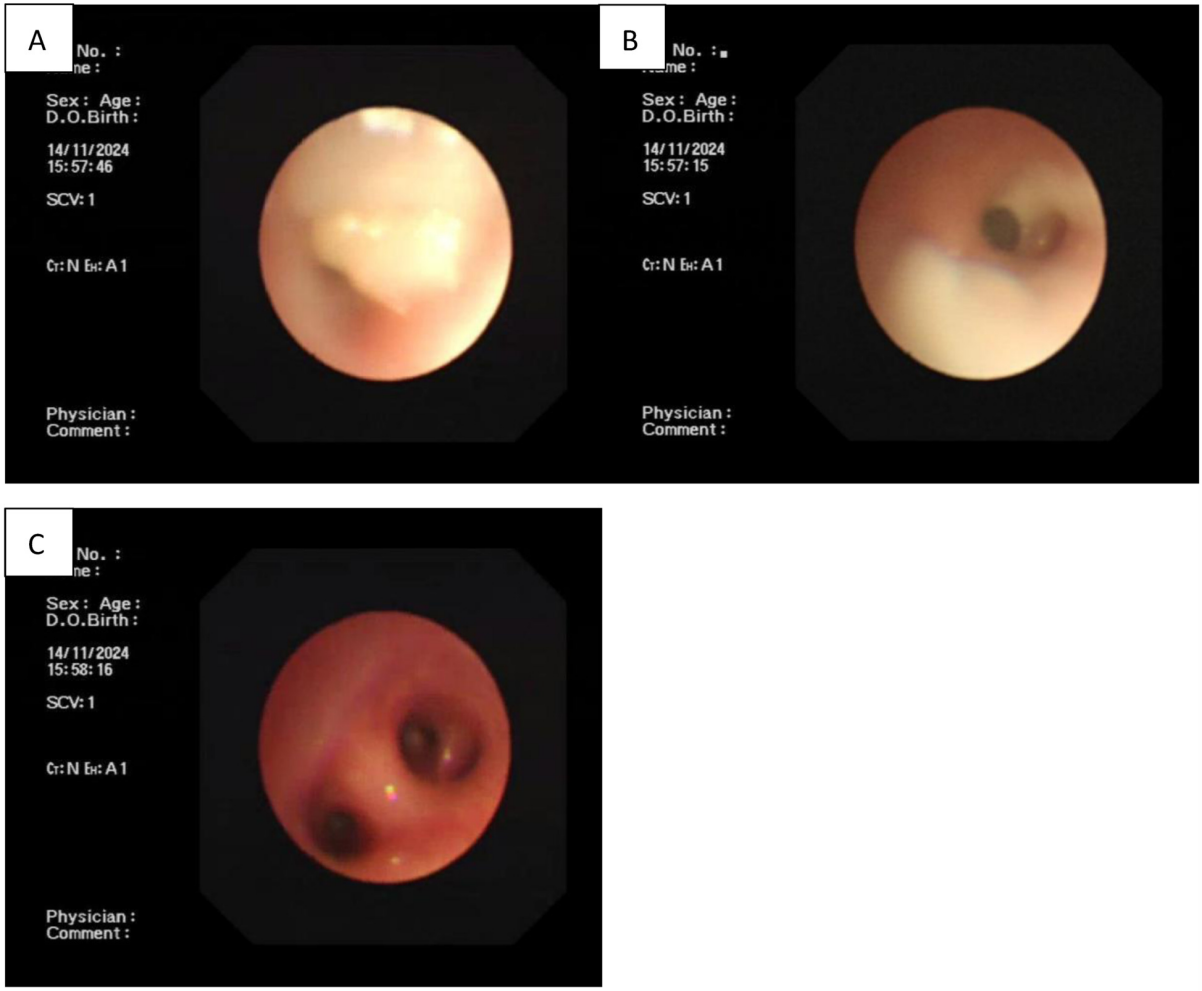
**(A**,**B)** Complete airway obstruction in the left upper and lower lobe bronchi as shown by electronic bronchoscopy. **(C)** Unblocked airway after bronchoscopic lavage treatment.

On the day of admission, deep sputum samples were collected via endotracheal intubation and analyzed using pathogen-targeted next-generation sequencing (tNGS). MP was the only pathogen detected; no other microorganisms were identified. Since the neonate had already been intermittently treated with azithromycin, no other antibiotics were administered; however, respiratory support was provided. After a 4-day interval, the second round of oral azithromycin was administered, combined with a low dose of methylprednisolone (2 mg/kg/day). On day 16 of life, the attempt to remove the tracheal tube failed and intubation was performed for the fourth time. Persistent respiratory distress with no signs of improvement raised concerns about a possible congenital airway anomaly in addition to MP infection. On day 21 of life, after extubation, electronic bronchoscopy was performed. There was no evidence of structural abnormalities in the airway; however, mucous plugs positive for MP were observed in the basal segments of the left upper and lower lobe bronchus. Bronchoscopic lavage was carried out, resulting in significant improvement in pulmonary ventilation (Figure 2). The day after electronic bronchoscopy, the neonate developed severe dyspnea and required a fifth intubation. Even after two full courses of azithromycin, the neonate remained dependent on ventilatory support.

Based on the mutation assay results of the macrolide-resistant MP gene at locus A2063G, the present case was classified as severe macrolide-resistant MP pneumonia. After consulting with the hospital’s Department of Pharmacy and obtaining parental consent, treatment with moxifloxacin was initiated at a dosage of 10 mg/kg/day on day 23 of life. Meanwhile, the methylprednisolone dose was increased to 4 mg/kg/day. On day 25 of life, chest radiography revealed marked improvement in left lung atelectasis (Figure 1B). On day 28, the neonate was successfully extubated but still required nasal CPAP, followed by gradual transition to oxygen via a nasal catheter. On day 33, lung function testing showed that both the peak time ratio and peak volume ratio were only slightly below the lower normal limits, while all other parameters remained within the normal reference ranges. On day 41, the neonate was weaned from off nasal oxygen support and was discharged with a diagnosis of mild bronchopulmonary dysplasia (BPD). Changes in routine blood tests and blood gas analysis from day 13 to day 33 of life are presented in Tables 1, 2, respectively.

**Table 1:** Changes in routine blood tests from the 13th to 33rd day of life.

Day of life	WBC (×10^9^/L)	Hb (g/L)	PLT (×10^9^/L)	NEUT (%)	LYMP (%)	MONO (%)	EOS (%)	CRP (mg/L)
13th	22.58	134	419	67.3	21.6	10.6	0.1	<1
15th	13.01	126	205	38.9	38.9	21.7	0.1	<1
19th	16.81	143	575	25.6	47.4	25.8	0.7	<1
21st	10.06	125	510	25.9	37.1	33.2	3.3	4.6
23rd	10.01	119	453	17.3	37.3	38.6	6.3	4.1
25th	11.28	103	678	16.9	39.4	40	3	1.58
33rd	6.28	103	328	15	57.3	19.4	7.5	<1

WBC, white blood cell count; Hb, hemoglobin; PLT, platelet count; NEUT, neutrophil; LYMP, lymphocyte; MONO, monocyte; EOS, eosinophil; CRP, C-reactive protein.

**Table 2:** Changes in blood gas examination from the 13th to 25th day of life.

Day of life	Oxygen supply status	PH	PO2 (mmHg)	PCO2 (mmHg)	Lactic acid (mmol/L)	BE value (mmol/L)	HCO_3_^−^ (mmol/L)
13th (other hospital)	TI	7.413	65.6	32.8	2.7	−3.6	22.2
13th (our hospital)	TI; OIC: 50%	7.415	51.1	37.2	2.9	−0.5	23.8
14th	TI; OIC: 50%	7.398	42.6	37	3	−1.6	22.8
15th	TI; OIC: 34%	7.426	42.5	38.1	1.4	0.8	25
16th	CPAP; OIC: 40%	7.338	50.1	47.3	1.2	−0.9	25.4
19th	TI; OIC: 38%	7.406	61.2	38.4	2.1	−0.4	24.1
20th	CPAP; OIC: 45%	7.271	50.5	59.1	1.6	−1	27.2
22nd	CPAP; OIC: 50%	7.358	42.8	51.5	1.2	2.5	28.9
25th	TI; OIC: 35%	7.38	51.6	46.1	1.5	2.1	27.6

TI, tracheal intubation; OIC, oxygen inhalation concentration; CPAP, continuous positive airway pressure; PH, pondus hydrogenii; PO_2_, oxygen permeance; PCO_2_; partial pressure of carbon dioxide; BE, base excess; HCO_3_^−^, hydrogen carbonate radical.

A 1-month follow-up was conducted after the neonate’s discharge, at a corrected gestational age of 41 weeks. His body weight was 3,350 g and length was 51 cm. He no longer required oxygen support, exhibited good feeding tolerance, and showed normal activity endurance. Given the diagnosis of BPD, ongoing evaluation of lung function was deemed necessary.

A subsequent follow-up was conducted 6 months after discharge. The infant was exclusively breastfed, with a length of 62 cm and weight of 5.5 kg. According to the Fenton growth curve, his length corresponded to the 15th–25th percentile, while his weight fell between the 1st–3rd percentiles for his age. Despite the suboptimal weight gain, the child did not require oxygen support, had no episodes of wheezing, tolerated feeding well, maintained normal activity levels, and showed completely normalized lung function upon re-examination. Due to the relatively low weight, enhanced nutritional support and ongoing monitoring height and weight were recommended.

## Discussion

MP is the leading cause of community-acquired pneumonia in children aged under 5 years in China. Unlike bacteria and viruses, MP lacks a cell wall and is the smallest prokaryotic microorganism capable of independent survival (6). In contrast to urogenital mycoplasmas—such as *Ureaplasma urealyticum*, *Mycoplasma genitalium*, and *Mycoplasma hominis*—MP primarily colonizes the urogenital tract (7–9). Reports of congenital MP pneumonia in neonates are rare, with this case representing only the fifth described in the literature. Congenital MP pneumonia typically presents with early-onset symptoms, as all five reported cases—including the present one—exhibited signs of dyspnea on the day of birth. It is possible that congenital MP pneumonia is attributed to maternal infection of MP during pregnancy, which may lead to chorioamnionitis via the placenta and cause ascending intrauterine infection. Consequently, affected neonates frequently experience preterm birth, low birth weight, and abnormal fetal development. Aside from one full-term newborn, four of the five cases of congenital MP pneumonia (four published plus the present case) involved premature infants. It is worth noting that this is the first reported case of congenital MP pneumonia with confirmed macrolide resistance treated with quinolones. It is also the first case diagnosed as severe congenital MP infection using electronic bronchoscopy.

Globally, particularly in East Asian countries, the resistance rate of MP to macrolide antibiotics is over 80%, and this resistance is extremely serious (10). The exact mechanisms behind the macrolide resistance are not fully understood, but are though to involve a combination of genetic, biochemical, and environmental factors that reduce bacterial susceptibility. Proposed mechanisms include genetic mutations, methylation changes, and drug inactivation (11). There is evidence that macrolide-resistant MP (*MRMP*) strains harbor point mutations in domain V of the 23S rRNA gene. Among these, the A2063G mutation is the most common (12–14). This mutation impairs the binding of macrolides to the 23S rRNA V domain of the 50S ribosomal subunit, preventing inhibition of peptide chain elongation and protein synthesis, and ultimately resulting in resistance (11). In the present case, the A2063G mutation was detected in the *MRMP* gene, underscoring the importance of genetic information in guiding clinical classification and medication.

In addition to macrolide resistance, the present neonatal case met the diagnostic criteria for severe MP pneumonia, as evidenced by persistent respiratory distress, repeated failures to wean from mechanical ventilation, and obvious pulmonary atelectasis. Prior studies have identified several risk factors associated with severe MP pneumonia, including advanced age, persistent fever, wheezing, atelectasis, macrolide resistance, and elevated levels of D-dimer, lactate dehydrogenase, C-reactive protein, and erythrocyte sedimentation rate (15–18). In this case, there was no evidence of persistent fever, possibly due to the early administration of dexamethasone aimed at preventing bronchopulmonary dysplasia. Although MP pneumonia predominantly affects preschool- and school-aged children, the risk of developing severe MP pneumonia has been shown to increase with age (18). In recent years, the incidence of severe MP pneumonia in children has shifted toward younger age groups. We hence suggest that more attention should be directed to the management of neonates congenitally infected with severe MP pneumonia to reduce the pediatric burden.

National guidelines recommend that macrolides, especially azithromycin, are the first-line treatment for mild MP pneumonia in children. In cases of severe MP pneumonia, tetracyclines are recommended for children aged ≥8 years, while macrolides remained the preferred choice for children aged <8 years. Fluoroquinolones are prescribed for off-label use in children under 18 years due to specific adverse effects, including tooth discoloration, enamel dysplasia, and cartilage damage (19). In the present case, the neonate failed to wean from ventilator support after two rounds of oral azithromycin, prompting the use of fluoroquinolone to combat the severe MP pneumonia. Despite the adverse effects, the off-label use of tetracyclines and fluoroquinolones in children has become more common, especially in the presence of macrolide resistance and severe MP pneumonia outbreaks.

The successful treatment of this case of congenital severe MP pneumonia was not solely attributed to the administration of fluoroquinolones, but also by the use of electronic bronchoscopy. Considering the neonate could not wean from the ventilator and that radiographs showed obvious atelectasis, bronchoscopy was prioritized as an alternative diagnostic intervention. The procedure revealed that the airway was blocked by several mucus plugs. After bronchoscopic lavage, pulmonary ventilation improved significantly, with no signs of atelectasis. Currently, there is no consensus regarding the timing for bronchoscopy in MP pneumonia. Although the majority of respiratory specialists recommend performing the procedure within 7–14 days of disease onset, others maintain that performing bronchoscopic lavage within 24 h of hospitalization can significantly shorten the length of hospital stay and reduce MP sequences (20). Further research is warranted to determine the ideal timing for bronchoscopy.

In summary, this severe neonatal case provides clinical evidence for the treatment of congenital MP infection complicated by atelectasis and macrolide resistance, for the administration of fluoroquinolones in proper dosages and courses, and for the adoption of bronchoscopic lavage as an effective treatment choice to clear airway obstruction and improve pulmonary ventilation.

## Data Availability

The original contributions presented in the study are included in the article/Supplementary Material, further inquiries can be directed to the corresponding authors.
